# The Neurosurgeon on Trial: A Scoping Review of International Trends in Medicolegal Litigation Related to Neurosurgery

**DOI:** 10.7759/cureus.107769

**Published:** 2026-04-26

**Authors:** Christopher Brian M Reyes, Rhoby U Orata, Abdul A Ontok

**Affiliations:** 1 Neurosurgery, Armed Forces of the Philippines Health Service Command – V. Luna Medical Center, Quezon City, PHL; 2 Surgery, Armed Forces of the Philippines Health Service Command – V. Luna Medical Center, Quezon City, PHL

**Keywords:** judicial settlements, lawsuit, malpractice, medicolegal, neurosurgery

## Abstract

The intersection of neurosurgery and legal issues has intensified due to reported increases in litigation rates in several regions, particularly in high-risk specialties such as spine surgery. This study investigates the trends and implications of medicolegal challenges in neurosurgery across various countries, emphasizing the need for a comprehensive understanding among practitioners and policymakers.

This scoping review followed the PRISMA-ScR guidelines. A literature search was conducted across databases, including PubMed, Scopus, and Web of Science, using key terms such as “neurosurgery,” “malpractice,” and “litigation,” focusing on peer-reviewed studies published within the past 15 years. Inclusion criteria targeted studies detailing claims specific to neurosurgeons, while excluding non-related literature. No formal quality appraisal of included studies was performed, consistent with the objectives of a scoping review.

Of the 180 identified studies, 13 met the eligibility criteria and collectively reported on 3,728 malpractice claims, predominantly from high-income countries, with 63% originating in the United States. While most cases favored neurosurgeons (61.29% overall), variations existed by region, reflecting differences in legal environments. Across the included studies, the distribution of reported claims over the study period appeared variable, with no consistent temporal trend identified. Notably, high monetary compensations were observed, with the U.S. averaging $439,146 per claim. These compensation figures are presented descriptively and do not imply comparative financial impact.

The review highlights the predominance of litigation associated with spinal procedures and describes the reported influence of defensive medicine on clinical decision-making. Despite favorable outcomes for neurosurgeons in low- to middle-income countries, the relatively lower number of reported claims may suggest possible underreporting in these settings. The findings underscore the need for improved documentation, communication strategies in clinical practice, and support systems for neurosurgeons. Continued research is necessary to better understand the relationship between medicolegal processes and patient care outcomes.

## Introduction and background

The practice of neurosurgery often interconnects with complex legal issues due to the high stakes involved in surgical interventions and patient outcomes. Over the past century, the legal landscape has become increasingly litigious due to various factors such as increased access to information, potential misinterpretation of medical data, advancements in medical technology, shifting public perception, and evolving healthcare and legal policies. Understanding these developments is necessary not just for neurosurgeons but also for legal professionals and policymakers to navigate the intricate relationship between medical practice and legal accountability. In this context, defensive medicine, defined as medical practices performed primarily to avoid litigation rather than to benefit the patient, has been increasingly recognized as a consequence of medicolegal pressures.

Globally, an increasing number of studies have highlighted concerns regarding the frequency and nature of litigation in neurosurgery. Several reports have indicated that the practice of neurosurgery, especially spine surgery, faces a higher risk of malpractice claims compared to many other specialties. This environment necessitates a thorough examination of these medicolegal issues, as they can significantly impact clinical decision-making and the overall practice of neurosurgery. Given the limited availability of local data, international evidence may provide important contextual insights that can inform understanding within the Philippine setting.

In the Philippines, medical malpractice suits in neurosurgery are complex and encompass administrative, civil, and criminal aspects. Administrative malpractice is regulated by the Medical Act of 1959, while civil and criminal cases fall under the Civil Code and the Revised Penal Code, respectively. Patients seeking damages must file civil actions based on Article 2176, which pertains to quasi-delict or civil negligence, while those pursuing criminal charges must reference Article 365.

Locally, there is still no published journal regarding medical malpractice lawsuits involving Philippine neurosurgeons. Concerning medical malpractice as a whole, Rebosa (2017) reported that one hundred twenty (120) lawsuits involving one hundred thirty-seven (137) physicians were identified between 2013 and 2014, with a noted upward trend [1]. These medical malpractice cases run across different specialties and include quasi-criminal, civil, and administrative proceedings.

The study of Fama (2022) reported 15 malpractice cases involving physicians from 2007 to 2017 based on Philippine Supreme Court proceedings. Among these cases, none of the defendants were neurosurgeons [2].

The importance of understanding trends in medical malpractice within neurosurgery extends beyond the individual practitioner. It encompasses systemic implications for hospitals and the legal framework governing medical practice. For instance, studies have shown that an increase in litigation rates can lead to defensive medicine practices, which may compromise patient care and inflate healthcare costs, such as through unnecessary diagnostic testing or interventions [3]. Therefore, a comprehensive analysis of international claims and reports is critical to identify patterns and inform strategies that enhance both patient safety and legal understanding in neurosurgery.

## Review

The review followed the methodological framework for scoping reviews provided by the Preferred Reporting Items for Systematic Reviews and Meta-Analyses extension for Scoping Reviews (PRISMA-ScR) checklist [4]. No formal critical appraisal or risk of bias assessment of included studies was performed, as the objective of this scoping review was to map the available evidence rather than evaluate study quality.

**Figure 1:**
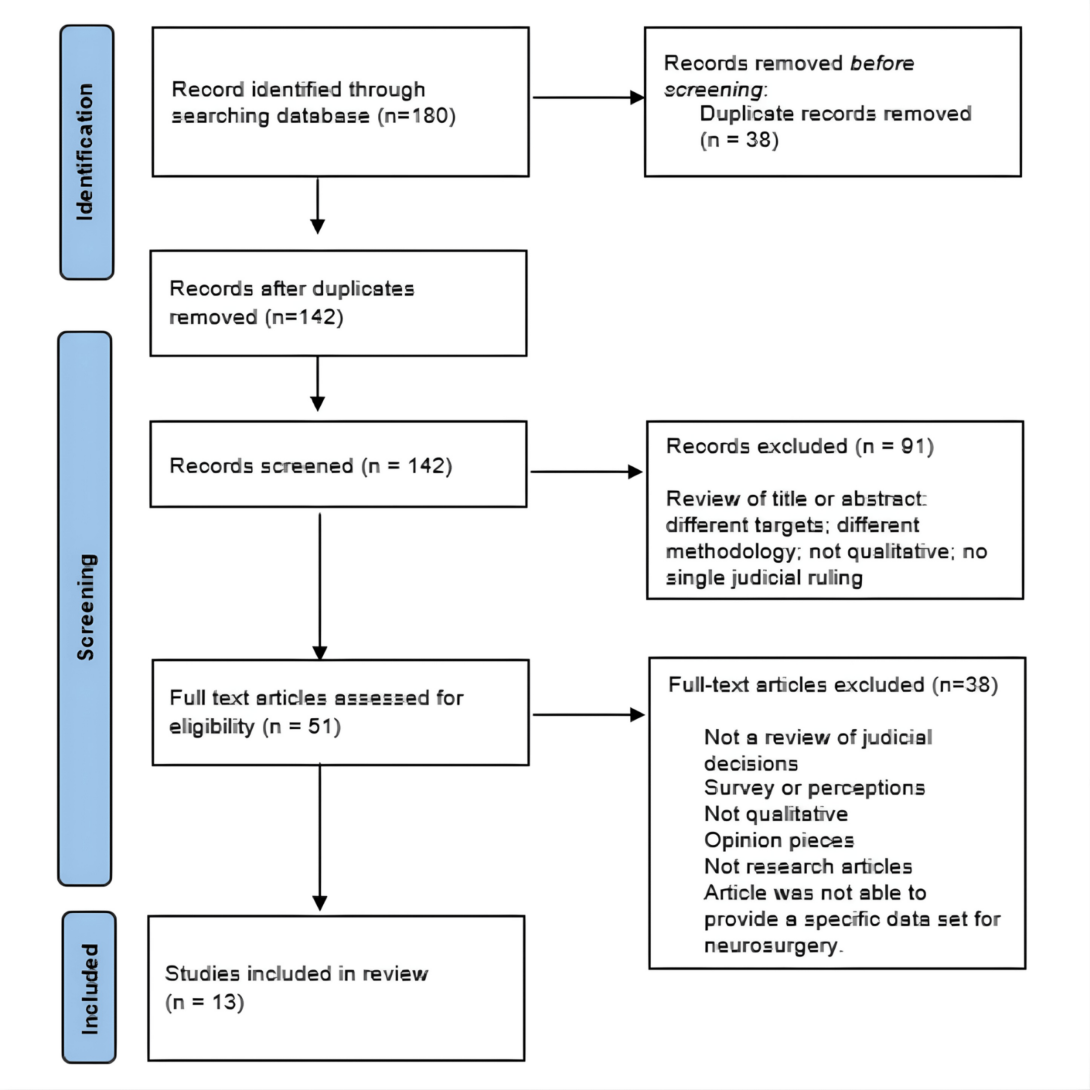
PRISMA-ScR flow diagram showing selection of sources of evidence PRISMA: Preferred Reporting Items for Systematic reviews and Meta-Analyses

A total of 180 records were identified through the search strategy. After the removal of 38 duplicate records, 142 records remained for title and abstract screening. Of these, 91 records were excluded. Fifty-one full-text articles were assessed for eligibility, of which 38 were excluded based on the predefined criteria. Thirteen studies were included in the final scoping review. The study selection process is summarized in Figure 1.

The studies reviewed are descriptive analyses of medical litigation involving neurosurgeons across various countries [5-17]. Among the included studies, 11 originated from high-income countries, while two studies, one from Brazil and one from Turkey, represented low- to middle-income countries (LMICs).

The studies collectively examine a total of 3,728 claims against neurosurgeons, with 63% originating from the United States. Notably, only 109 of these cases (2.9%) come from low- to middle-income countries (LMICs). This distribution demonstrates a higher concentration of reported litigation in high-income regions, particularly in the United States.

Specifically, the research identifies four studies from Asia contributing 194 claims, one study from Brazil with 79 claims, six studies from Europe totaling 1,099 claims, and two studies from the United States accounting for 2,356 claims. Table 1 presents the detailed characteristics of the included studies, including country of origin, number of cases reviewed, data sources, compensation information, and key findings.

**Table 1:** Included studies are arranged by authors, publication year, geographic location of the research, key findings, and conclusions and recommendations

Author; Publication Year	Country;	Number of Cases Reviewed against Neurosurgeons	Time Frame and Setting	Data Source	Monetary Compensation involved	Key Findings
de Macêdo Filho, L. J. M.; Aragão, A. C. A.; Moura, I. A.; Olivier, L. B.; Albuquerque, L. A. F. 2020 [5]	Brazil;	79 cases	January 2008 to February 2020 Private and Public Hospitals	Brazilian Hospital Information System (Sistema de Informações Hospitalares do Sistema Único de Saúde [SIHSUS]) records JusBrasil platform	The average compensation awarded per case was reported to be about fifteen times higher than the usual median professional fee for a neurosurgical procedure, was ($145.28*) *Php 8,356.50 at USD = 57.52 PhP	Court decisions were unfavorable to neurosurgeons in 26.58% of cases. Spine-related procedures accounted for the majority of lawsuits, while cases involving brain tumors were associated with the highest compensation amounts.
Emery, E.; Balossier, A.; Mertens, P. 2014 [6]	France	116 cases were noted (34 Cranial and 81 Spinal Procedures was convicted)	1997-2007 Public Hospitals	Data bank of the insurer Société Hospitalière d’Assurances Mutuelles (SHAM, main insurance company for public hospitals in France)	Data not disclosed.	In cranial cases, 97% of rulings were unfavorable to neurosurgeons. All documented spine cases resulted in conviction. The most common reasons cited were surgical site infection (37%), technical errors (22%), inadequate patient information (14%), delayed diagnosis (11%), and lack of supervision (9%)
Samara, E.; Tzoumas, L.; Tzoumas, K.; Lenas, A.; Papadopoulos, G. 2022 [7]	Greece	52 cases	1985-2021	Published court decisions of criminal, civil, administrative and disciplinary content were searched in Greek legal information banks	The average compensation was 101 701€ ^+ ^€6,339,0230 at €1 = 62.33 PhP	In the cases included in the study 5/52 cases (9.61%) resulted in an acquittal. Majority of the cases was from a spine case.
Nagashima, H.; Wada, Y.; Hongo, K. 2017 [8]	Japan	46 cases (Dataset included 446 for all specialties)	January 2001 – December 2015	No Central Database used. The healthcare-related negligence lawsuits were retrieved and sorted in each clinical field from the database in different Courts in Japan	Data not disclosed.	Among the neurosurgical cases reviewed, 26 were found to involve negligence, while 12 were dismissed. The identified issues included diagnostic errors, problems in clinical judgment, technical deficiencies, management concerns, and inadequate informed consent. In more recent cases, deficiencies in the informed consent process were repeatedly noted.
Otsuki K, Watari T. 2020 [9]	Japan	95 cases	1961 - 2017	National Japanese malpractice claims database	The adjusted median indemnity paid was $234,997* *13,515,874 PhP at USD = 57.52 PhP	In malpractice claims involving neurosurgeons, 62.1% resulted in payment. Wrong diagnosis occurred in 25 (69.4%) cases. Delayed diagnosis occurred in 4 cases and 7 missed diagnosis cases. Stroke was the most common presenting condition, with subarachnoid hemorrhage accounting for a significant proportion of total indemnity payouts
Dronkers, Wouter J.; Amelink, Quirine J. M. A.; Buis, Dennis R.; Broekman, Marike L. D.; Spoor, Jochem K. H. 2020 [10]	Netherlands	57 cases (All cases were won by the plaintiff. Dismissed cases are confidential) (1322 neurosurgical care related cases)	2009-2019	Website of the Dutch Disciplinary Court for Medical Professionals’ records using Dutch neurosurgery-related search terms between November 2019 and February 2020	Not disclosed but sanctions may be imposed on an individual healthcare professional, including a warning, a reprimand, a fine of up to 4500€^+^, a temporary suspension, or a permanent revocation of a medical license ^+ ^€280,485 at €1 = 62.33 PhP	Most complaints involved spine surgery (62.5%), followed by cranial procedures. Issues were mainly related to preoperative and intraoperative care.
Shim, J.-H.; Lee, K.-S.; Shim, J.-J.; Yoon, S.-M.; Bae, H.-G.2012 [11]	South Korea	23 cases	1978-2010	Precedents in the Korean Supreme Court web site	Not disclosed in the study.	Eleven cases were determined to involve negligence. Court decisions did not appear to correlate with court level, patient outcome, principal issue, or treatment modality. Trends over time suggested shifting patterns in liability attribution across decades.
Hernández-Herrero, M.; Cayón de las Cuevas, J. 2022 [12]	Spain	38 cases	2008-2020	High of Justice (Administrative Courts)	The median award was 40 000€^+^, with a range of 5000–78 285€ ^+ ^€2,493,200 at €1 = 62.33 PhP	More than half of first-instance rulings were dismissed (51.85%), increasing to 88.88% at the appellate level. Lack of adequate patient information was the leading basis for compensation awards (53.33%). Sequelae represented the most frequently claimed harm (81.57%), with spinal surgery comprising the largest proportion of cases (60.52%).
Gundogmus, U. N.; Erdogan, M. S.; Sehiralti, M.; Kurtas, O. 2005 [13]	Turkey	30 cases against neurosurgeons (997 cases for all cases)	1993-1998	The cases reported to the Higher Health Council in Turkey	Not disclosed in the study.	43% (9/30) of the cases have noted that the neurosurgeons were liable for the cases
Hamdan, A.R. D. S.; Strachan, F.; Nath, I. C.; Coulter, I. C.2015 [14]	England, United Kingdom	794 claims	2002 - 2012	National Health Service Litigation Authority	The mean claim per successful case of £160,000^#^ ^#^12,011,878 PhP at £1 = 75 PhP	A total of 66.1% of claims were dismissed. Spinal procedures were the most litigated (44.1%). The predominant cause of claims was substandard surgical performance (29.1%). Claims involving wrong-site surgery and cauda equina syndrome frequently resulted in convictions.
Mukherjee, S.; Pringle, C.; Crocker, M.2013 [15]	St George Healthcare NHS Thrust, United Kingdom	42 claims	March 2004 – March 2013	Risk management system of St George Healthcare NHS Thrust, United Kingdom	The highest median payouts were for claims against faulty surgical technique (£230,000) and delayed diagnosis/misdiagnosis (£212,650) ^#^12,011,878 PhP at £1 = 75 PhP	Twelve claims (28%) were unfavorable to neurosurgeons. The majority of cases were resolved outside court. Spinal procedures predominated. Frequent allegations included technical errors (43%), delayed or missed diagnosis (17%), inadequate patient information (14%), and treatment delay (12%).
Kessler, R. A.; Benzil, D. L.; Loewenstern, J.; Chen, S.; Bhammar, A.; Kohli, K. M.; Hadjipanayis, C. G.; Scarrow, A.; Bederson, J.; Shrivastava, R. K.2019 [16]	36 states and Territories in the United States	225 cases	1985-2016	WestLawNext, a prominent legal database in the United States	Not discosed in the study	In jury-decided cases, defendants prevailed in approximately 60% of instances. The most common allegations involved failure to diagnose and failure to treat. Pituitary adenoma and acoustic neuroma were the most frequently cited conditions.
Elsamadicy, A. A.; Sergesketter, A. R.; Frakes, M. D.; Lad, S. P. 2018 [17]	United States	2131 cases	2003-2012	Physician Insurers Association of America Data Sharing Project	The average indemnity for neurosurgery claims $439 146. The largest neurosurgery claim payment during this time period was $5 600 000,	Among 2,131 claims, 28.06% resulted in indemnity payment. Improper performance was the leading contributing factor (42%). Intervertebral disc disorder was the most common presenting diagnosis (20.6%). A total of 22.91% of claims involved patient death.

Dynamics of court decisions for neurosurgeons

This synthesis provides an overview of legal cases involving neurosurgeons across 13 studies from various countries, totaling 3,728 cases, as shown in Table 2. The outcomes reveal variation in the proportion of cases favoring plaintiffs and neurosurgeons across different regions.

**Table 2:** A quantitative summary of the cases presented in the study

Author	Country;	Number of Cases	Cases favorable to the plaintiffs	%	Cases favorable to Neurosurgeons	%
de Macêdo Filho, L. J. M.; Aragão, A. C. A.; Moura, I. A.; Olivier, L. B.; Albuquerque, L. A. F. [5]	Brazil;	79	21	26.58	58	73.42
Emery, E.; Balossier, A.; Mertens, P. [6]	France	116	113	97.41	3	2.59
Samara, E.; Tzoumas, L.; Tzoumas, K.; Lenas, A.; Papadopoulos, G. [7]	Greece	52	47	90.38	5	9.62
Nagashima, H.; Wada, Y.; Hongo, K. [8]	Japan	46	26	56.52	20	43.48
Otsuki K, Watari T [9]	Japan	95	59	62.11	36	37.89
Dronkers, Wouter J.; Amelink, Quirine J. M. A.; Buis, Dennis R.; Broekman, Marike L. D.; Spoor, Jochem K. H. [10]	Netherlands	57	57	100	0	0
Shim, J.-H.; Lee, K.-S.; Shim, J.-J.; Yoon, S.-M.; Bae, H.-G. [11]	South Korea	23	11	47.83	12	52.17
Hernández-Herrero, M.; Cayón de las Cuevas, J. [12]	Spain	38	18	47.37	20	52.63
Gundogmus, U. N.; Erdogan, M. S.; Sehiralti, M.; Kurtas, O. [13]	Turkey	30	9	30	21	70.00
Alhafidz Hamdan, R. D. S.; Strachan, F.; Nath, I. C.; Coulter, I. C. [14]	United Kingdom	794	389	48.99	405	51.01
Mukherjee, S.; Pringle, C.; Crocker, M. [15]	United Kingdom	42	12	28.57	30	71.43
Kessler, R. A.; Benzil, D. L.; Loewenstern, J.; Chen, S.; Bhammar, A.; Kohli, K. M.; Hadjipanayis, C. G.; Scarrow, A.; Bederson, J.; Shrivastava, R. K. [16]	United States	225	93	41.33	132	58.67
Elsamadicy, A. A.; Sergesketter, A. R.; Frakes, M. D.; Lad, S. P. [17]	United States	2131	598	28.06	1533	71.94
-	Total	3728	1453	38.98	2275	61.02

In France and the Netherlands, interpretation of outcomes may be limited due to potential reporting bias, as dismissed cases are often not publicly disclosed due to confidentiality.

In contrast, Brazil showed a more balanced outcome, with 26.58% of cases favoring plaintiffs and 73.42% favoring neurosurgeons. Similarly, Turkey demonstrated strong support for neurosurgeons, with 70% of cases ruled in their favor, despite only a small number of total cases.

The United Kingdom and the United States presented favorable results for neurosurgeons across the included studies. In one United Kingdom study, 51.01% of cases favored neurosurgeons, while another indicated a higher 71.43% in favor of neurosurgeons. In the United States, a total of 2,356 cases showed that a majority of cases favored neurosurgeons across datasets.

Japanese studies presented varied results, with one study showing 43.48% favorability for neurosurgeons and another at 37.89%.

Overall, the data illustrate variation in outcomes by country. On a broader scale, 61.02% of cases overall favored neurosurgeons.

Monetary compensation is involved in the claims

The data on monetary compensation in neurosurgery malpractice cases reveal variation across countries. In Brazil, as an LMIC, the mean compensation per procedure is relatively low at $145.28, although this represents a substantially higher value relative to the median professional fee for neurosurgical procedures. Greece presents a more straightforward picture with an average compensation of €101,701. Meanwhile, Japan's adjusted median indemnity of $234,997 reflects reported compensation values, despite incomplete disclosure across all studies.

The Netherlands emphasizes regulatory measures over specific compensation data, while the UK shows a mean claim ranging from £160,000 to £230,000 with notable payouts for specific malpractice categories. The United States reports the highest average indemnity for neurosurgery claims at $439,146, with some claims reaching up to $5,600,000.

These findings are presented descriptively and reflect differences in reporting practices and legal systems across the included studies.

Factors that cause the plaintiff’s claims

The analysis of malpractice claims against neurosurgeons across various countries reveals critical trends and commonalities that illuminate the challenges within the field. The prevailing theme is the high incidence of litigation associated with spinal procedures, as evidenced by findings from Brazil, Greece, the Netherlands, and the UK, where spine surgeries consistently generated the majority of lawsuits. This trend suggests a need for enhanced focus on this subspecialty to improve patient communication and expectations.

In Brazil, the brain tumor subspecialty incurred the highest compensation, highlighting the financial stakes involved in neurosurgical litigation. Meanwhile, France identified significant causes for malpractice, with surgical site infections and technical errors leading the list. This aligns with findings from the UK, where inadequate surgical performance was the most common claim, emphasizing the importance of surgical technique and operational protocols.

Japan's data revealed alarming rates of misdiagnosis and issues surrounding informed consent, with negligence cited as a fundamental cause of litigation. The implications from Japan has noted that improving diagnostic accuracy and ensuring thorough patient communication are essential for minimizing legal exposure. This data is congruent with Spain and Turkey, where the lack of information frequently upheld compensation claims.

Discussion

The study was among the first to synthesize available evidence using a scoping review approach on medicolegal claims pertaining to neurosurgery across various countries. It was noted that although a majority of claims favor neurosurgeons, existing literature suggests that medicolegal concerns may contribute to stress and burnout among practitioners. In the United States, the reported burnout rate among neurosurgery residents reached 67% [18]. This is supported by a study by Gadjradj et al. (2020), in which 39.5% of respondents reported being frequently or always concerned about litigation, while 77.4% reported that this concern influenced their clinical practice, and 12.4% reported avoiding complex cases due to fear of litigation [19].

Fear of litigation has been associated with the practice of defensive medicine, defined as a deviation from standard medical practice motivated by the desire to reduce exposure to malpractice liability [20].

Defensive medicine has been associated with increased utilization of imaging, laboratory tests, and referrals that may not directly benefit the patient. Such practices may contribute to delays in appropriate treatment and increased healthcare utilization.

While previous studies have estimated the economic impact of defensive medicine, such estimates were not derived from the included studies and are therefore not the focus of this review.

In low- to middle-income countries (LMICs), although they accounted for only 2.9% of the total claims, 79 out of 109 cases (72%) favored neurosurgeons. This finding may reflect differences in reporting systems, legal processes, or access to medicolegal mechanisms, although further investigation is required.

The analysis of monetary compensation highlights variation across studies and settings, which may be influenced by differences in reporting practices and legal frameworks. Given the heterogeneity of the included studies, direct comparisons across countries should be interpreted with caution.

The prevalence of litigation, particularly in spine procedures, underscores the importance of effective communication between neurosurgeons and patients to manage expectations and reduce misunderstandings that may lead to claims. Furthermore, concerns regarding litigation may influence clinical decision-making and contribute to changes in practice patterns.

These findings suggest the importance of strengthening clinical communication, documentation, and support systems for healthcare providers. However, conclusions regarding policy or systemic reforms should be interpreted cautiously, given the descriptive nature of the data.

Continued research is necessary to further clarify the relationship between medicolegal processes and clinical practice.

This study has several limitations. First, the included studies were heterogeneous in terms of methodology, study period, and reporting standards, which limits direct comparability. Second, the reliance on published databases and legal records may introduce selection bias and underreporting, particularly in regions with limited documentation systems. Third, only selected databases were used, which may have excluded relevant studies from other sources. Fourth, no formal quality appraisal was conducted, consistent with the objectives of a scoping review, but this may affect the interpretability of the findings. Finally, variations in legal frameworks and healthcare systems across countries further limit the generalizability of the results.

## Conclusions

This scoping review provides a comprehensive analysis of the medicolegal landscape surrounding neurosurgery, highlighting trends in malpractice litigation across various countries. The findings indicate a substantial number of reported claims, particularly in spine surgery, although a majority of cases result in favorable outcomes for neurosurgeons. While the profession faces medicolegal challenges, existing literature suggests that these concerns may influence clinical decision-making and contribute to the practice of defensive medicine.

Differences in reported monetary compensation across countries reflect variation in reporting practices and legal systems, although direct comparisons should be interpreted with caution due to heterogeneity among studies. The review highlights the importance of improving communication between neurosurgeons and patients, particularly in managing expectations and strengthening informed consent processes. Although low- to middle-income countries report fewer cases, this may be influenced by differences in reporting systems or access to medicolegal processes. Given the descriptive nature of the available data, conclusions regarding systemic or policy-level implications should be interpreted cautiously. Continued research is needed to better understand the relationship between medicolegal processes, clinical practice, and practitioner well-being.
